# The Effect of Sugar-Free Versus Sugar-Sweetened Beverages on Satiety, Liking and Wanting: An 18 Month Randomized Double-Blind Trial in Children

**DOI:** 10.1371/journal.pone.0078039

**Published:** 2013-10-22

**Authors:** Janne C. de Ruyter, Martijn B. Katan, Lothar D. J. Kuijper, Djin G. Liem, Margreet R. Olthof

**Affiliations:** 1 Department of Health Sciences and the EMGO Institute for Health and Care Research, Faculty of Earth and Life Sciences, VU University, Amsterdam, The Netherlands; 2 School of Exercise and Nutrition Sciences, Centre for Physical Activity and Nutrition Research, Deakin University, Burwood, Victoria, Australia; University of Missouri-Kansas City, United States of America

## Abstract

**Background:**

Substituting sugar-free for sugar-sweetened beverages reduces weight gain. A possible explanation is that sugar-containing and sugar-free beverages cause the same degree of satiety. However, this has not been tested in long-term trials.

**Methods:**

We randomized 203 children aged 7-11 years to receive 250 mL per day of an artificially sweetened sugar-free beverage or a similarly looking and tasting sugar-sweetened beverage. We measured satiety on a 5-point scale by questionnaire at 0, 6, 12 and 18 months. We calculated the change in satiety from before intake to 1 minute after intake and 15 minutes after intake. We then calculated the odds ratio that satiety increased by 1 point in the sugar-group versus the sugar-free group. We also investigated how much the children liked and wanted the beverages.

**Results:**

146 children or 72% completed the study. We found no statistically significant difference in satiety between the sugar-free and sugar-sweetened group; the adjusted odds ratio for a 1 point increase in satiety in the sugar group versus the sugar-free group was 0.77 at 1 minute (95% confidence interval, 0.46 to 1.29), and 1.44 at 15 minutes after intake (95% CI, 0.86 to 2.40). The sugar-group liked and wanted their beverage slightly more than the sugar-free group, adjusted odds ratio 1.63 (95% CI 1.05 to 2.54) and 1.65 (95% CI 1.07 to 2.55), respectively.

**Conclusions:**

Sugar-sweetened and sugar-free beverages produced similar satiety. Therefore when children are given sugar-free instead of sugar-containing drinks they might not make up the missing calories from other sources. This may explain our previous observation that children in the sugar-free group accumulated less body fat than those in the sugar group.

**Trial Registration:**

ClinicalTrials.gov NCT00893529 http://clinicaltrials.gov/show/NCT00893529

## Introduction

Recent trials have shown that sugar-free beverages lead to less weight gain than sugar-sweetened drinks[1,2]. A possible explanation is that sugars in solution are detected incompletely by receptors that determine satiation. As a result, sugar-free and sugar-containing drinks should produce similar degrees of satiety[3], and intake of calories from other foods is not affected[4]. Previous studies indeed showed that masked replacement of sugar-containing beverages with sugar-free beverages was only partly compensated for by an increased energy intake[5,6]. Small experiments lasting a few days[7,8,9], or a few weeks[10,11] showed that satiety following sugar-containing and sugar-free artificially sweetened drinks was similar. However, data from large, long-term double blind trials are lacking, especially in children. 

Liking and wanting are food rewards that also influence food intake[12]. Liking reflects the immediate experience or anticipation of pleasure from eating a food, i.e. the hedonic value or ‘palatability’ of the food. Wanting is the intrinsic motivation to engage in eating a food, now or in the (near) future[13]. A four-week study in adults found that sugar-free beverages were preferred less than sugar-containing beverages[10], but another study failed to confirm this[11]. Again, large long-term trials are lacking. 

We therefore investigated satiety, liking and wanting in DRINK, a double-blind randomized controlled trial in which children replaced their habitual daily sugar-containing drink with either a sugar-free or sugar-sweetened beverage for 18 months[1,14]. 

## Methods

### Ethics statement

Written informed consent was provided by a parent or guardian who had obtained assent from the child. The Medical Ethical Committee of VU University Medical Centre Amsterdam approved the study protocol. The protocol for this trial and supporting CONSORT checklist are available as supporting information; see Checklist S1 and Protocol S1.

### Study population

**Figure 1:**
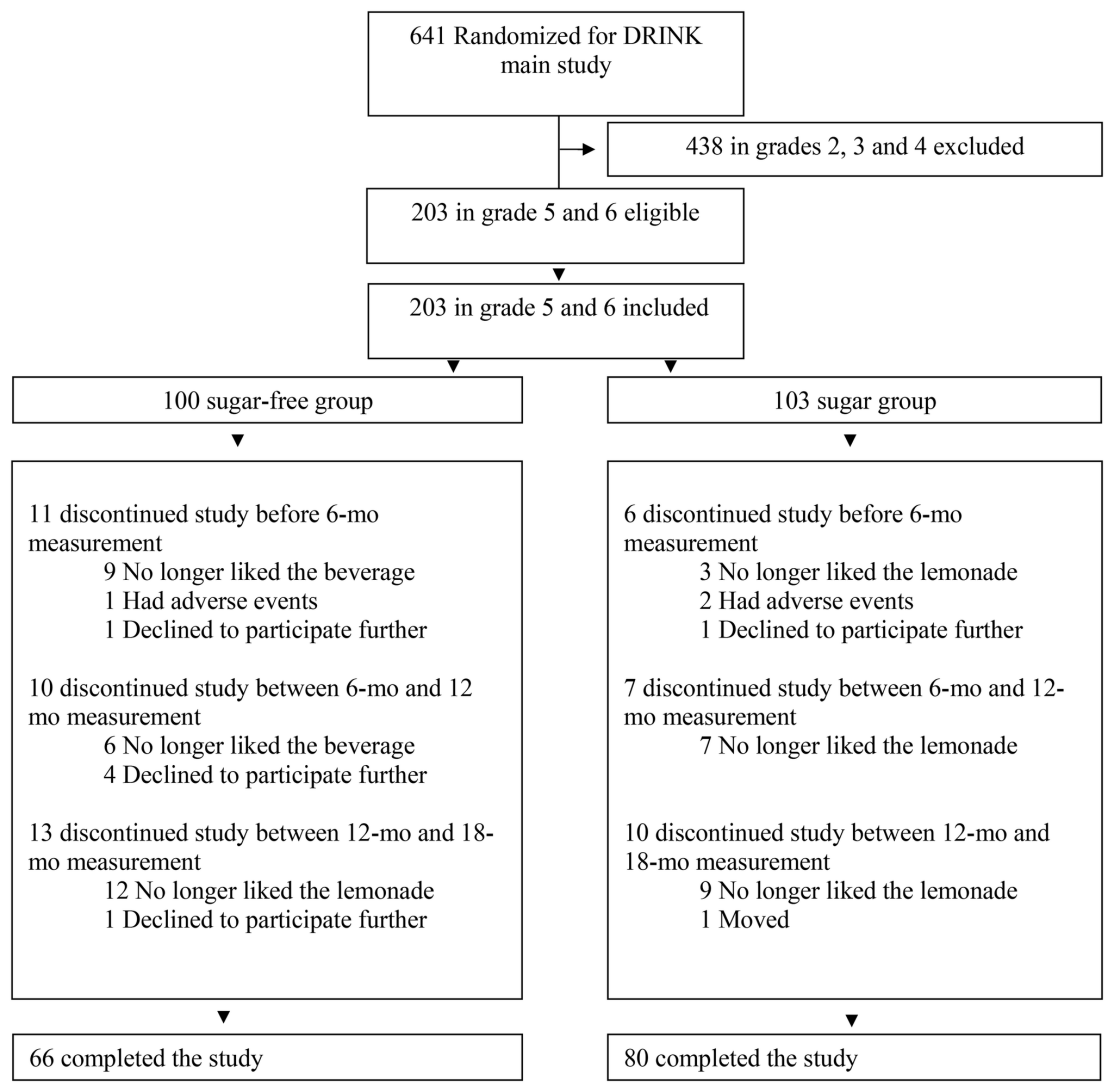
Randomization and follow-up of the study participants. In the sugar-free group, allergy was reported as an adverse event for one child. In the sugar-group, allergy was reported as an adverse event for one child, and weight increase was reported as an adverse event for one child.

The primary objective of the DRINK trial was to examine the effect of masked replacement of sugar-sweetened beverages with noncaloric, artificially sweetened beverages on weight gain. The design and results have been reported[1,14]. Here we report effects on satiety, liking and wanting. DRINK was an 18-month double-blind randomized controlled trial in 641 children aged 5-11 living near Amsterdam, the Netherlands. Participants were individually randomized to receive either 250 ml per day of a sugar-free, artificially sweetened beverage with 0 kcal (sugar-free group) or a similar sugar-sweetened beverage that provided 104 kcal (sugar group). For the present study, only participating children in the two highest school grades were eligible because young children would not be able to understand the questionnaire. All 203 of them proved willing and able to participate, and were enrolled (Figure 1). We recruited participants between August and November 2009[14]. Treatment started between November 14 and December 7, 2009. The trial lasted 19.5 months. We interrupted treatment for 1.5 months during the summer holidays of 2010. 

### Study beverages

Dutch primary school children habitually bring a snack and a beverage to school for the morning break. We replaced the beverage brought from home with our study beverages. We provided children with 1 can per day of a noncaloric, artificially sweetened, noncarbonated beverage or a sugar-sweetened noncarbonated beverage. We developed custom drinks for this study to ensure that the sugar-free and sugar-sweetened drinks tasted and looked essentially the same. The identical-looking 250-ml cans provided either 0 or 26 g of sucrose (0 or 104 kcal per day). The sugar-free beverages contained 34 mg sucralose and 12 mg acesulfame K as sweeteners. After the DRINK trial of 18 months the large majority of children were unable to guess whether they had received sugar-sweetened or sugar-free drinks[1]. Each week children received a box at school labelled with their name and containing 8 cans, 1 for each day of the week plus 1 extra to be used as a spare in case a can was misplaced. We offered beverages in four flavours: raspberry, peach, lemon and mango. Flavours were rotated every two weeks. 

### Satiety, liking and wanting

**Figure 2:**
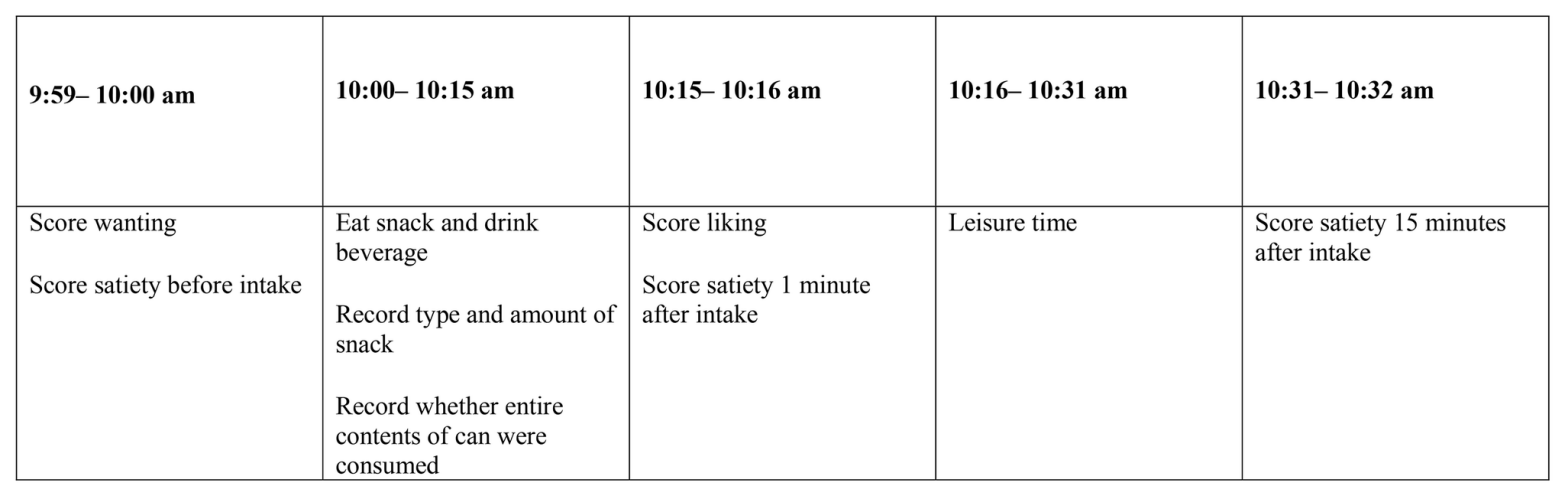
Time schedule for filling out the questionnaire. The timeline is indicative because each school had its own timing of the morning break.

We measured satiety, liking and wanting with a questionnaire (Figure 2; Supporting information, Appendix S1). The satiety scale has been validated in children aged 4-6 years old who indicated satiety produced by three different imagined eating situations[15]. Children showed different outcomes for each meal suggesting that our satiety scale indeed measures satiety. Leon et al. compared three methods to measure liking in 169 children aged 4 to 10 years old[16]. They concluded that the scale used by us provides a reliable and valid estimate of liking in children aged 8-10 years. The wanting scale has been used earlier [17] but has not been formally validated yet.

 Children completed a single questionnaire (of satiety, liking and wanting) on one day every 6 months. They did this during the 10:00 am morning break when they consumed their study drink and snack. The teachers instructed the children explicitly how to fill out the questionnaire and to consume their drink. The teacher also asked them to write down in the questionnaire whether they had finished the contents of the can. One researcher was present at each school and circulated between school classes during the measurements. The children filled out the questionnaires themselves without assistance.

 We measured satiety, liking and wanting on 5-point scales; 1 indicated low levels, and 5 high levels (Supporting information, Appendix S1). We measured satiety three times on each test day: just before intake of the study beverage and the food brought from home, 1 minute after intake, and 15 minutes after intake[3]. We measured wanting 1 minute before and liking 1 minute after intake. The questionnaire also inquired about snack consumption, i.e. the amount and type of food eaten with the study beverage. We calculated the calories in these snack food[18,19]. 

### Statistical Analyses

We present outcome variables as medians and interquartile ranges. We used generalized linear mixed model analysis in STATA SE 12 (StataCorp LP, Texas, USA). The generalized linear mixed model analyses took into account the repeated measurements at 0, 6, 12 and 18 months, and the ordinal structure of the 5-point scales. For children who discontinued beverage intake we included the data obtained up until discontinuation. 

 We calculated regression coefficients for the likelihood that on the 5-point satiety scale the sugar-group increased by 1 point more than in the sugar-free group. We separately calculated increases in satiety from before intake to 1 minute after intake, and from before intake to 15 minutes after intake by adjusting for satiety prior to intake (model 1). In model 2 we additionally adjusted for caloric intake from snacks with the beverage, for gender and for BMI z score. Adjustments for gender and BMI z score served to correct for imbalances in randomization. We used similar analyses to calculate differences between treatment groups in liking and wanting of the beverages. Calculations of liking and wanting were adjusted for gender and BMI z score at the start of the study. We also calculated regression coefficients for effect modification by month of measurement. The sugar-free group always served as the reference group. Therefore, odds ratios larger than 1 imply that the effect in the sugar group was larger than that in the sugar-free group, and vice versa. We also present arithmetic means of scores to show the differences between groups, and how the means changed with time. However, we did not perform statistical analyses of the arithmetic means because the outcomes were on an ordinal scale. We used the conventional limits for significance of P = 0.10 for effect modification and P = 0.05 for all other analyses. 

## Results

### Participants

The 203 participants were aged 10.2 (0.8) years at the start of the study, mean (SD). Baseline characteristics were similar for the two treatment groups, except that the sugar group had more girls, a lower mean BMI, and parents had on average achieved higher education levels (Table 1). A total of 146 (72%) children completed the study, 66 in the sugar-free group and 80 in the sugar group (Figure 1). Of the 57 children who stopped drinking the beverages, 46 or 81% did so because they no longer liked the beverages. 

**Table 1:** Baseline characteristics of participants.^a^

**Characteristic**	**Sugar-free group (N = 100)**	**Sugar group (N = 103)**
Girls (%)	42%	52 %
Age (years)	10.2 (0.73)	10.2 (0.80)
Dutch ancestry ^b^	89%	92%
Non-western ancestry	10%	7%
Parent education lower to intermediate ^c^	20%	8%
Parent education high school	23%	25%
Parent education college or university	56 %	66%
Weight (kg)	36.86 (6.14)	36.36 (6.51)
Height (cm)	144.4 (7.28)	145.5 (7.49)
Body-mass index^d^	17.6 (2.2)	17.1 (2.2)
Body-mass index z score (SD units above Dutch mean) ^e^	0.02 (0.99)	-0.09 (0.90)
Low body-mass index ^f^	6%	3%
Healthy body-mass index	78%	85%
Overweight	16%	12%
Obese	0%	0%
Sum of four skinfolds (mm)	41.5 (16.7)	37.8 (17.4)
Waist-to-height ratio (%)	43.6 (3.9)	42.3 (3.6)
Electrical-impedance fat mass (kg) ^g^	7.5 (3.2)	6.9 (3.3)
Electrical-impedance fat mass (% of body weight)	20%	18%

^a^ Values are means (SD) or percentages, as indicated.

^b^ N = 201; 2 households did not fill out this form. A child is designated *Dutch* if both parents were born in the Netherlands, and *Non-western* if one or both parents were born in Suriname, Dutch Antilles, Turkey, Morocco, Russia, Egypt or Vietnam.

^c^ N = 201; 2 households did not fill out this form. *Lower to intermediate education* is Elementary school, Lower vocational secondary education, Technical secondary education, Intermediate secondary education or Intermediate vocational education. We based educational level on whichever of the parents had the highest education.

^d^ Body-Mass Index Is the Weight in Kilograms Divided by the Square of the Height in Meters

^e^ We calculated z score of body-mass index and height from the Dutch 2009 reference data[32].

^f^ We used international cut-offs for low and healthy body-mass index [33] and for overweight and obesity._ENREF_31[34]

^g^ N = 202; 1 child refused the measurements

### Study beverage and food intake

The percentage of children who consumed the entire content of the can in the morning when questionnaires were applied ranged from 87% to 98% across the duration of the trial. These percentages were similar for the two groups (Table 2). Cross contamination and compliance were described previously[1]. The can counts showed that 85% of the cans in both groups had been consumed. Analysis of sucralose in urine showed that children in the sugar-group had not consumed sugar-free beverages. The caloric intake from the snacks brought from home was 137 (90) kcal in the sugar-free group and 140 (84) kcal in the sugar group, mean (SD) (Table 2)[18,19]. Most children (77%) brought sweet snacks such as crackers, cookies, cakes, or bars, 20% brought fruits, 2% brought savoury snacks such as cheese and chips, and 1% brought bread.

**Table 2:** Medians with interquartile ranges of satiety, liking, wanting, and beverage- and snack intake, measured on a 5-point scale in children.^a^

**Variable**	**Sugar-free Group**	**Sugar Group**		**Sugar-free Group**		**Sugar Group**		**Sugar-free Group**	**Sugar Group**		**Sugar-free Group**	**Sugar Group**	
Month of measurement													
	**0 Mo**		**0 Mo**	**6 Mo**			**6 Mo**	**12 Mo**		**12 Mo**	**18 Mo**		**18 Mo**
	**N=95**		**N=99**	**N=86**			**N=95**	**N=75**		**N=88**	**N=63**		**N=78**
Satiety before intake	1 (1 to 2)		2 (1 to 2)	2 (1 to 3)			2 (1 to 2)	2 (1 to 2)		2 (1 to 2.75)	2 (1 to 3)		2 (1 to 2)
Satiety 1 minute after beverage intake	3 (2 to 4)		3 (2 to 4)	3 (3 to 4)			3 (2 to 4)	3 (2 to 4)		3.5 (2 to 4)	3 (2 to 4)		3 (2 to 4)
Satiety 15 minutes after beverage intake	2 (1 to 3)		2 (2 to 3.25)	2 (2 to 3)			2 (2 to 3.5)	2 (1 to 3)		3 (2 to 4)	3 (2 to 4)		3 (2 to 4)
Liking of the beverage	4 (3 to 5)		5 (4 to 5)	4 (3 to 4)			4 (3 to 4)	3.5 (3 to 4)		3 (3 to 4)	3 (2 to 4)		3 (2 to 4)
Wanting of the beverage	4 (4 to 5)		4 (4 to 5)	4 (3 to 4)			4 (3 to 5)	3 (2 to 4)		3 (2.25 to 4)	3 (2 to 4)		3 (2 to 4)
Percentage of children who drank entire can	95%		98%	98%	97%			87%		89%	93%		88%
Caloric intake from snack (kcal) consumed with the study beverage, mean (SD)	129.2 (81.9)		131.9 (77.3)	149.6 (102.3)	144.4 (87.7)			127.4 (76.2)		139.7 (87.0)	142.9 (97.3)		147.2 (83.4)

^a^ Values are median (interquartile range, x to x) or mean (SD) unless otherwise notes. Numbers of participants do not match those in Figure 1 because of absentees.

### Satiety

**Figure 3:**
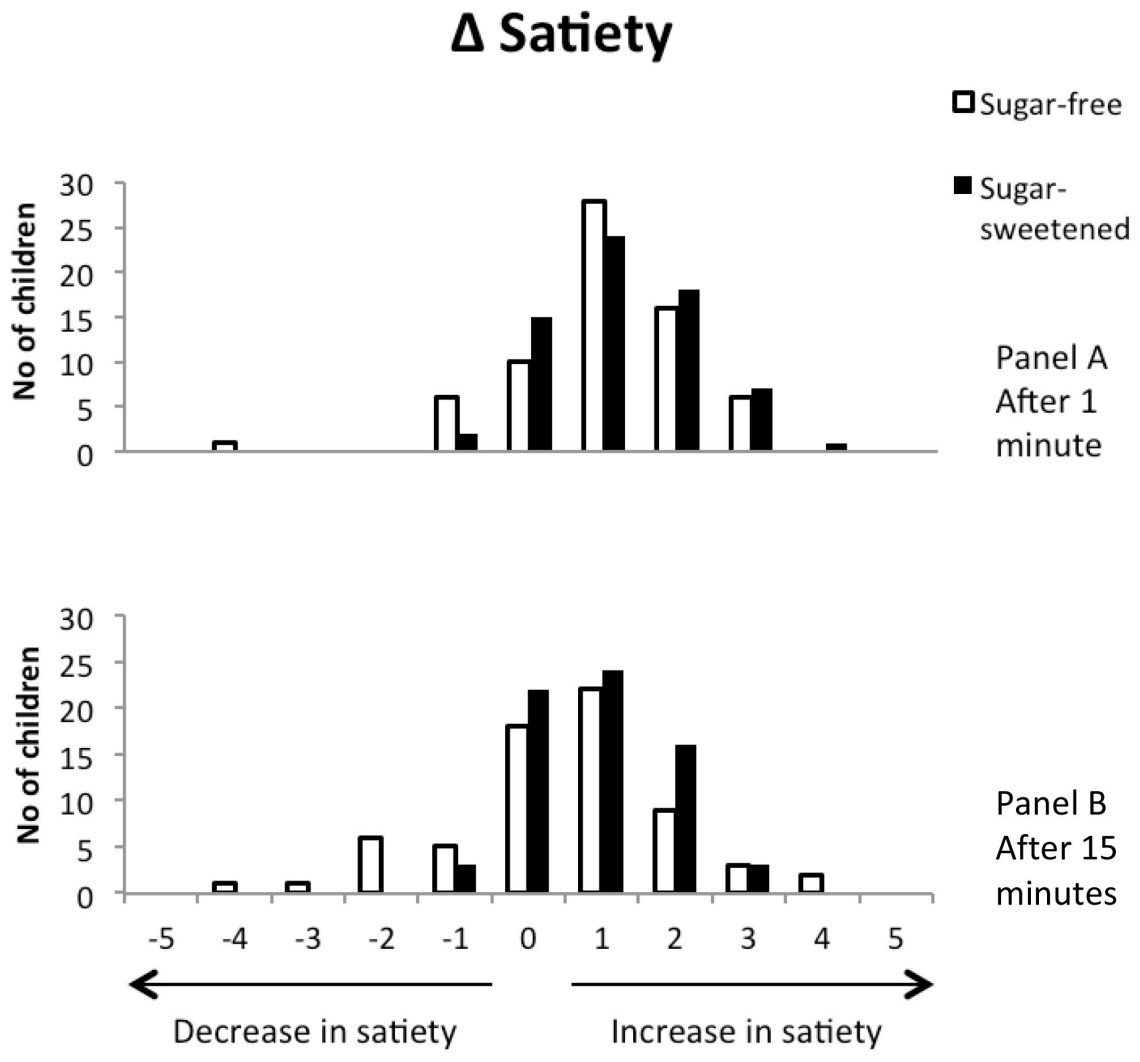
Satiety after intake minus satiety before intake of the beverages at 18 months in 146 children. Satiety was scored from 1 (not full at all) to 5 (very full). Bars indicate the shift in satiety from before to after intake of the beverages. Panel A shows satiety 1 minute after intake; Panel B, 15 minutes after intake. An increase of 1 means any increase of 1 point on the 5-point scale.

**Figure 4:**
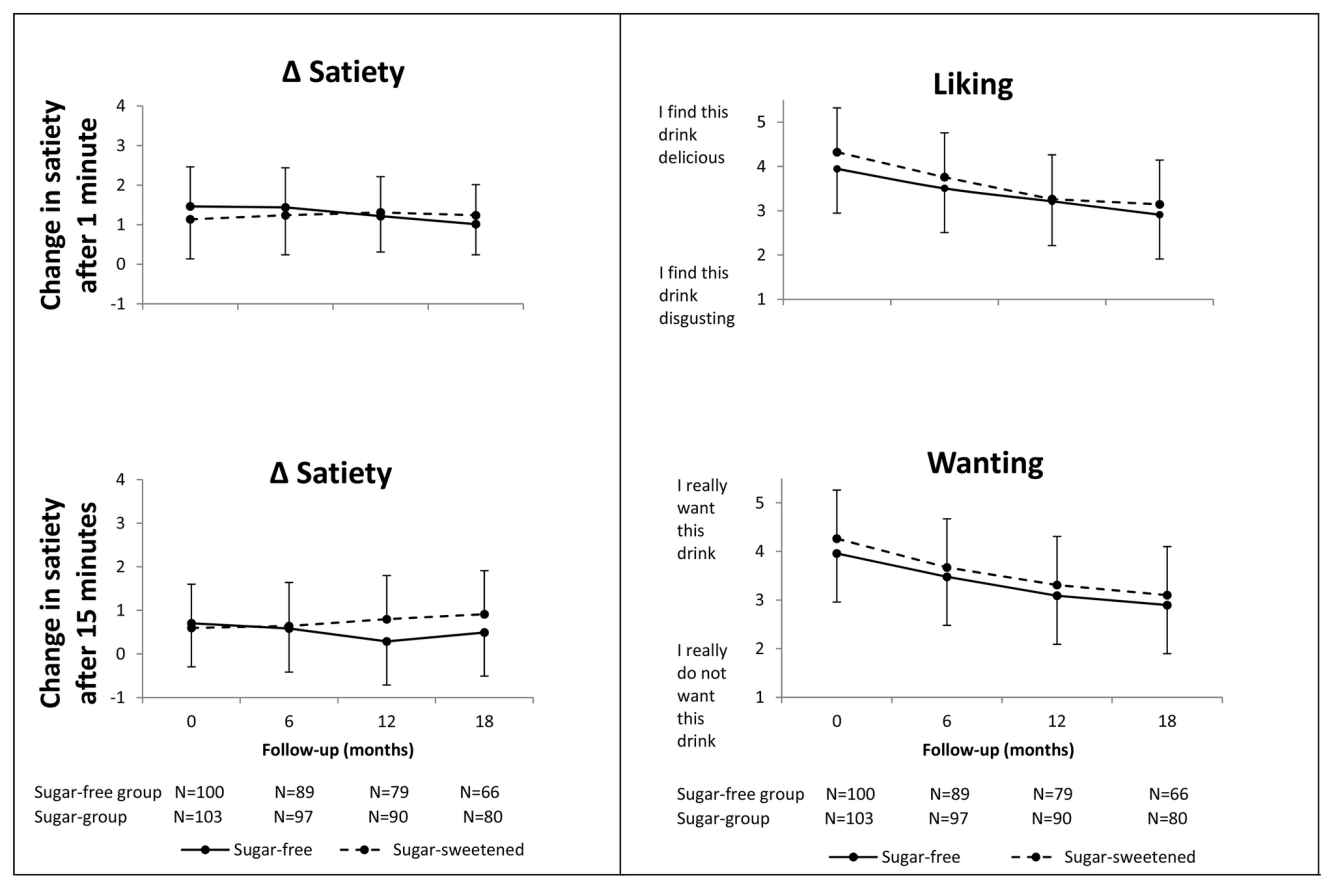
Arithmetic mean scores for satiety, liking, and wanting over the course of the trial. Dashed lines, sugar group; solid lines, sugar-free group. T bars indicate one standard deviation

In both groups, satiety was lowest before and highest 1 minute after beverage intake, and then decreased during the subsequent 14 minutes (Table 2; Figure 3). Sugar content of the beverages did not significantly affect satiety (Table 3; Figure 4). At 1 minute after intake, the odds ratio for satiety was 0.84 which implies that the likelihood of satiety to increase by 1 point was 0.84 times as high in the sugar group as in the sugar-free group (Table 3). At 15 minutes, the effect was in the opposite direction, with an odds ratio of 1.49, but again this was not statistically significant. Adjustments for caloric intake from the snack, for gender and for BMI z score at the start of the study had only minor effects on outcomes (Table 3). Effect modification by timepoint, i.e. month of measurement, was not statistically significant 1 minute after intake (P = 0.53), but marginally significant 15 minutes after intake (P=0.09).

**Table 3:** Odds ratios for the effect of sugar-sweetened versus sugar-free beverages on satiety, liking and wanting across the duration of the trial.

	**Sugar-group Odds ratio (95% CI**)	**P for difference between groups**
**Satiety 1 minute after beverage intake ^a^**		
Model 1 ^b^	0.84 (0.50 to 1.40)	0.50
Model 2 ^c^	0.77 (0.46 to 1.29)	0.33
**Satiety 15 minutes after beverage intake**
Model 1 ^b^	1.49 (0.89 to 2.49)	0.13
Model 2 ^c^	1.44 (0.86 to 2.40)	0.16
**Liking ^d^**		
Model 1 ^e^	1.58 (1.02 to 2.47)	0.04
Model 2 ^f^	1.63 (1.05 to 2.54)	0.03
**Wanting**
Model 1 ^e^	1.59 (1.03 to 2.46)	0.04
Model 2 ^f^	1.65 (1.07 to 2.55)	0.02

^a^ Odds ratios indicate the likelihood that satiety in the sugar group increased by 1 point more than satiety in the sugar-free group.

Odds ratio < 1 indicates less, and odds ratio >1 indicate more satiety in the sugar group than in the sugar-free group

^b^ Adjusted for satiety prior to the beverage intake

^c^ Adjusted for satiety prior to the beverage intake, snack intake, gender, and baseline BMI z score

^d^ Odds ratios indicate the likelihood that liking or wanting were 1 point higher in the sugar-group than in the sugar-free group.

Odds ratio < 1 indicates less, and odds ratio >1 indicate more liking or wanting in the sugar group than in the sugar-free group

^e^ Crude model

^f^ Adjusted for gender and baseline BMI z score

### Liking and wanting

The children randomized to receive sugar-sweetened beverages liked these slightly more than those who received sugar-free beverages for 18 months; the odds ratio for a difference of 1 point on the liking scale was 1.58 (Table 3). However, when the scores on the 5-point scale were averaged arithmetically the differences between the group means were small, and much smaller than the overall fall in liking over the course of the trial (Figure 4). Average liking fell by 1.18 points in the sugar group and by 1.04 points in the sugar-free group, while the difference between groups was 0.4 (1.06) points at baseline and 0.2 (1.24) points at 18 months, mean (SD) (Figure 4). Effect modification by month of measurement was not statistically significant (P= 0.27). This suggests that duration of consuming the drinks did not influence the differences in liking between groups. The children also wanted sugar-sweetened beverages more than sugar-free beverages; the odds ratio for a 1-point difference was 1.59 (Table 3; Figure 4). Again, effect modification by month of measurement was not statistically significant (P= 0.97).

## Discussion

We found that sugar-free and sugar-sweetened beverages produced similar degrees of satiety in a large population of children who consumed such beverages daily for 18 months. We previously reported that the children who consumed the sugar-free beverages accumulated less body fat than children who consumed sugar-sweetened beverages[1]. The present study suggests that this may be explained by similar levels of satiety. When we substituted sugar-free beverages for the sugar-containing drinks that children drank habitually, they apparently did not feel a need to eat more of other foods and drinks to compensate for the missing calories.

We tested satiety under real-life conditions, i.e. during the morning break at school when children consumed their beverage together with their usual snack. We assume that satiety was determined by snack intake – mostly crackers, sweets, or fruits - , beverage volume, and caloric content of the drink. Mean caloric intake from snacks and volume of drinks were the same between groups but the sugar-free drinks contained 26 g less sucrose. We conclude that the sugar content of the drinks did not have a measurable effect on satiety. This finding is consistent with short term experiments in adults[7,8,9,10,11] that also found similar levels of satiety following sugar-containing and sugar-free beverages. Effect modification by month of measurement for satiety 15 minutes after intake was marginally significant. This could indicate that sugar-sweetened beverages became more satiating as the trial proceeded. However, this may have been a chance finding.

We found that the children liked and wanted the sugar-sweetened beverage slightly more than the sugar-free beverage. These differences persisted throughout the study even though overall liking and wanting of both types of beverage fell drastically with time. In contrast, short-term studies with pudding[20], or beverages[11] found similar ratings of pleasantness for aspartame-sweetened and sucrose-sweetened products. However, beverages containing a blend of aspartame, acesulfame K and saccharin were rated lower in pleasantness than beverages containing sucrose[10]. Another study reported that cream cheese sweetened with aspartame had a more pleasant taste than cream cheese containing stevia or sucrose[21]. Thus the pleasantness of artificially sweetened products may be highly dependent on the type and mix of sweeteners used and on other aspects of product formulation. According to the literature different neural pathways are involved in liking and wanting[12]. However, our data suggest that these concepts may be related since they produced similar results. Future research is needed to examine whether wanting and liking indeed involve the same neural pathway or that our wanting scale lacked validity.

Liking and wanting of the trial drinks decreased markedly in both groups over the course of the trial. Similar declines have been reported in studies with solid foods over periods of 15 days to 6 months[22,23,24]. The decrease in liking and wanting agrees with our observation that most children who discontinued intake gave dislike of the beverage as their reason. A year and a half is indeed a long time for a child to drink the same drink every day, and the variety of flavours that we offered was obviously not enough to overcome this. Sensory-specific satiety may have decreased liking and wanting over time[25]. 

Our study had several strengths. One is its long duration. Previous studies had a maximum duration of 4 weeks[10]. Long term studies may be more informative because short term satiety signals may have little to do with the long term mechanisms that determine weight gain. Our study was large; we included 203 subjects, as opposed to 11 to 42 in previous studies[7,8,9,10,11]. Also, we used a double-blind design that eliminated the effects of psychological cues and socially desirable behaviour. Previous studies were either incompletely blinded[7,9] or not blinded[8]. Finally, we performed our study in children while previous studies investigated adults only. Regulation of food intake in adults may differ from that in children[26,27].

Our study also had limitations. Of the 203 participants, 57 children or 28% did not complete the study. However, we found similar results when the data of these 57 children were left out (data not shown). The lack of a statistically significant effect does not exclude that we may have failed to pick up small differences in satiety between beverage groups. However, since the effects were in opposite directions at 1 and 15 minutes after intake, we consider it likely that sugar-free and sugar-sweetened beverages did not lead to systematically different levels of satiety. The dietary status of our children was less standardized than in previous studies which included an overnight fast plus a standardized breakfast[8,9]. Finally, we measured satiety by questionnaire and we did not quantitate actual food intake following the beverage intake[7,8,9].

 The participants in our study were healthy Dutch children, and most of them were white and of normal weight. Future studies should be done to find out whether our findings hold for other ethnic groups or for obese children. Future studies may also examine the effect on satiety at intervals longer than 15 minutes[28]. 

 One important question is whether our findings are unique for sugars in liquid form. Short term studies with semi-solid foods such as pudding and jelly suggested that noncaloric sweeteners produced the same degree of satiety as sucrose[20,29]. It is unknown whether (covert) removal of solid calories is detected by receptors that determine satiety, although there is evidence that such removal reduces satiety and leads to compensatory intake of calories from other sources[30,31]. 

 We found that sugar-sweetened and sugar-free beverages produced similar satiety. Therefore when children are given sugar-free instead of sugar-containing drinks they might not make up the missing calories from other sources. This may explain our previous observation that the children in the sugar-free group accumulated less body fat than those in the sugar group[1].

## Supporting Information

Checklist S1 **CONSORT checklist.**
(DOCX)

Protocol S1 **Study protocol including amendments.**
(PDF)

Appendix S1 **Sensory questionnaire with English translation.**
(DOCX)
