# Supplementary material for: The Effect of Sugar-Free Versus Sugar-Sweetened Beverages on Satiety, Liking and Wanting: An 18 Month Randomized Double-Blind Trial in Children
Source: PLoS One. 2013 Oct 22;8(10):e78039. doi: 10.1371/journal.pone.0078039 (PMC3805601; doi:10.1371/journal.pone.0078039)
Supplement: Appendix S1 — Sensory questionnaire with English translation. (DOCX) [file pone.0078039.s003.docx]

Appendix 1: Sensory questionnaire in Dutch.

For this appendix we added the English translation in italics.

VOORNAAM *(Name)*:
ACHTERNAAM *(Last name)*:
GROEP *(Grade)*:
SCHOOL *(School)*:


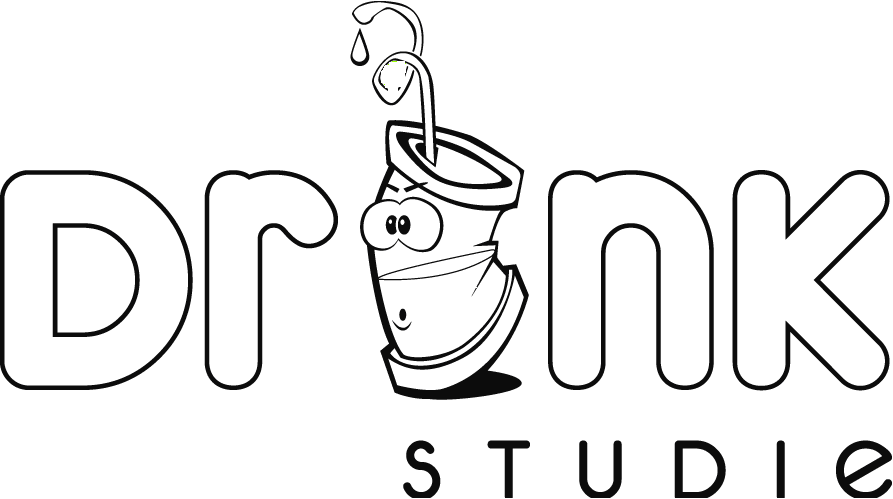


VRAGENLIJST *(Questionnaire)*

Hoi!

Voor je ligt de DRINK-studie vragenlijst. De vragen gaan over wat je van Blikkie vindt. Bij het invullen gaat het erom dat je laat weten hoe **JIJ** je voelt. Er is hierbij geen goed of fout. Als er staat dat je Blikkie op mag drinken, dan eet en drink je rustig zoals je gewend bent, voordat je de volgende vragen invult.

Als je bij de **RODE BLADZIJDE** bent, **STOP** je met invullen en na de pauze vul je de laatste bladzijde in. Na het invullen kan je de vragenlijst bij je juf of meester inleveren.

Alvast bedankt!

*Hi!*

*In front of you is the DRINK study questionnaire. The questions are about what you think of Blikkie. When you fill in the questionnaire we would like to know how you are feeling. There are no incorrect or correct answers. When you come across ‘Time to eat & drink’, you first eat and drink as you normally would. You then fill in the questions.*

*When you see a red page, stop. You now go outside, and after the break, you fill in the last question.*

*When you are finished, please give the questionnaire to the teacher.*

*Thanks*

**Hoeveel zin heb je in het drankje? *(****Do you feel like drinking the beverage?)*

**Voor dat je Blikkie proeft** willen wij graag weten hoeveel zin je **NU** in **BLIKKIE** hebt. Je kruist een hokje aan achter het blikje waarvan jij denkt dat bij jou past

*(Before you taste Blikkie, we would like to know if you feel like drinking Blikkie.You tick the box that matches what you feel)*


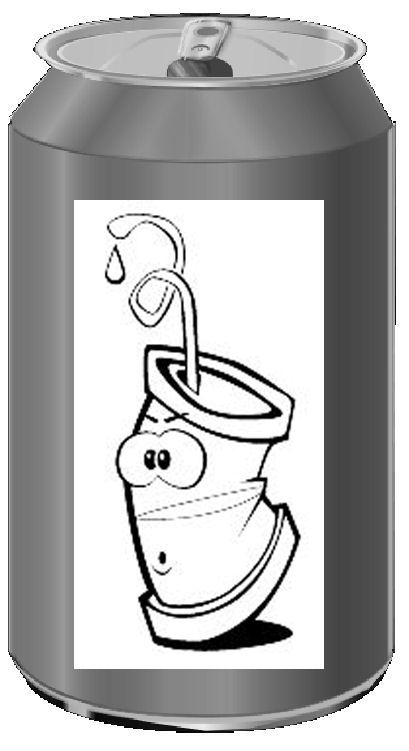

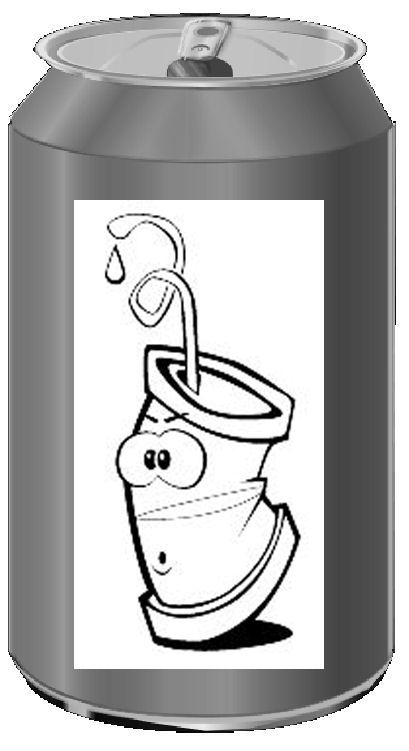

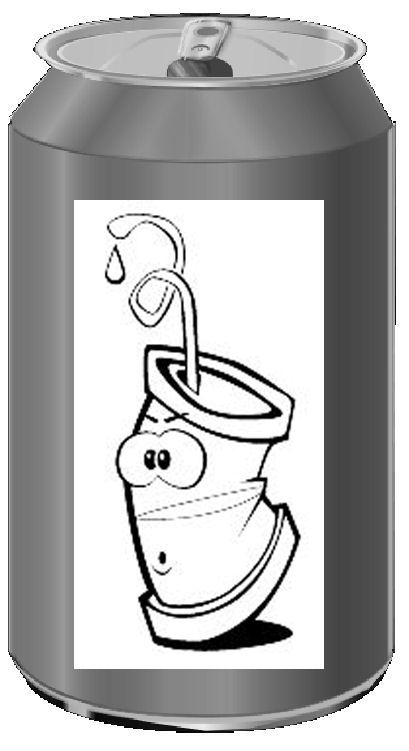

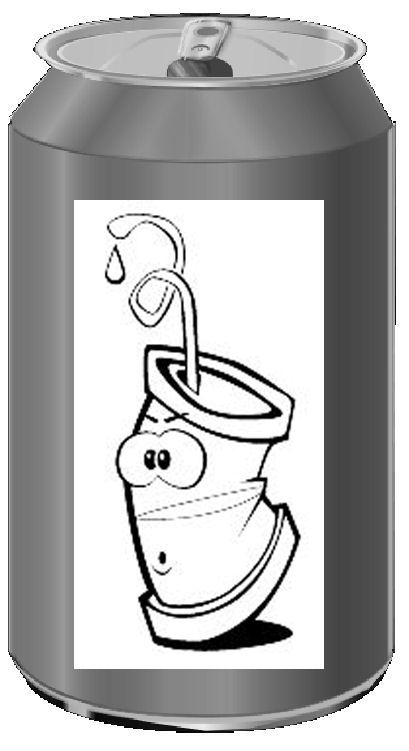

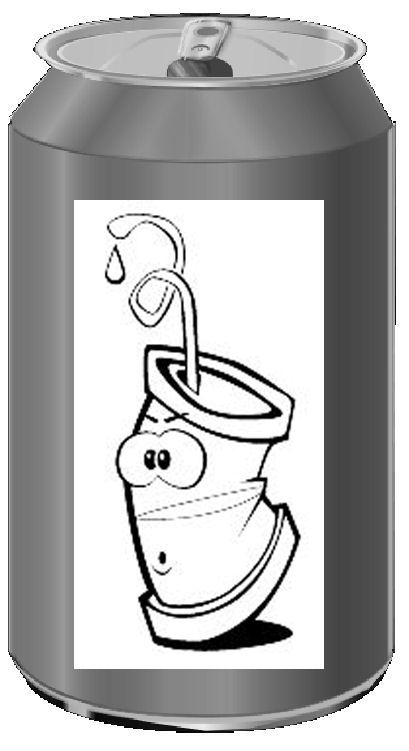

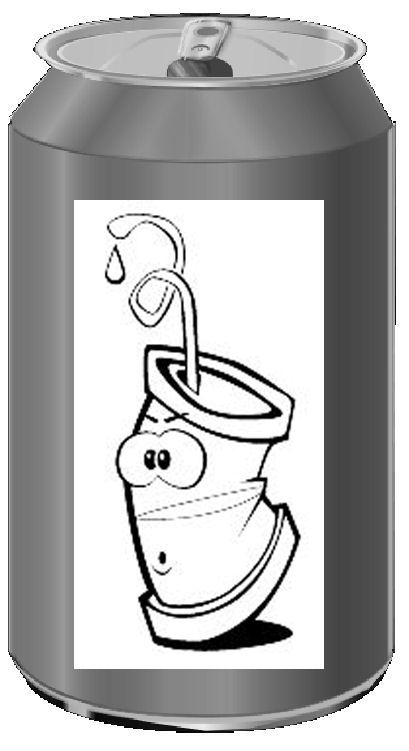

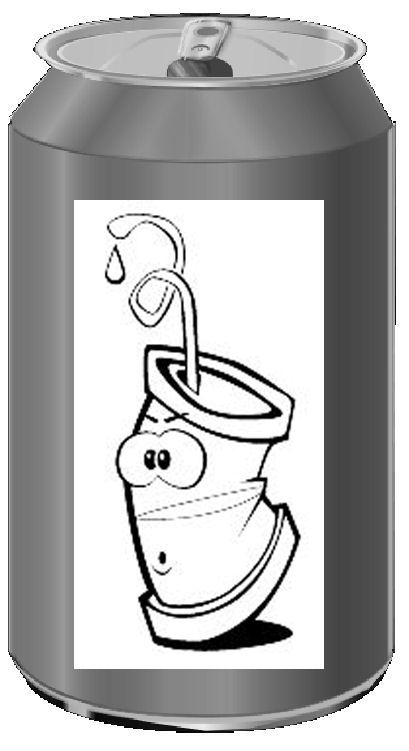

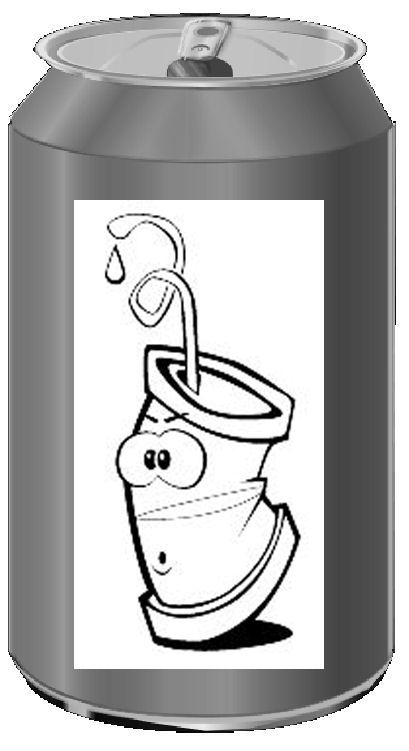

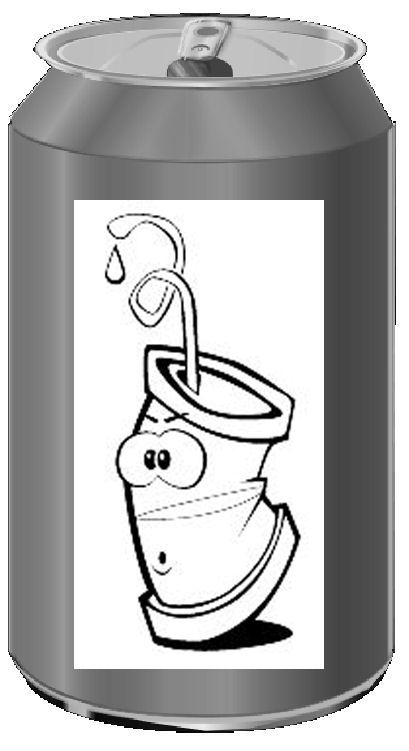

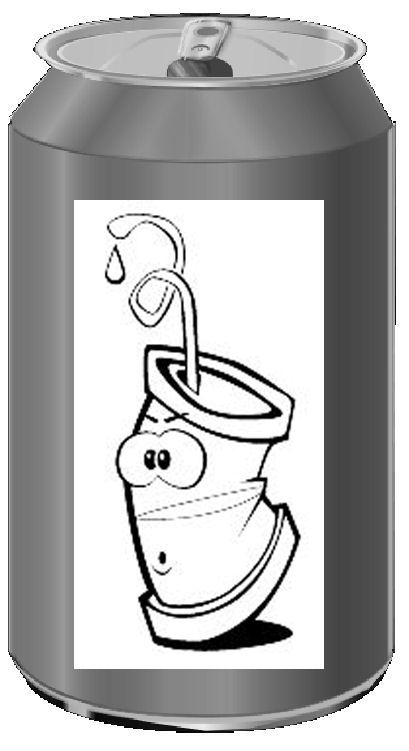

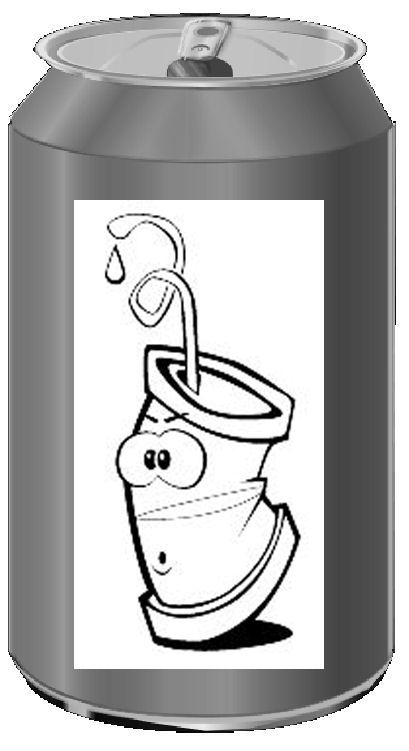

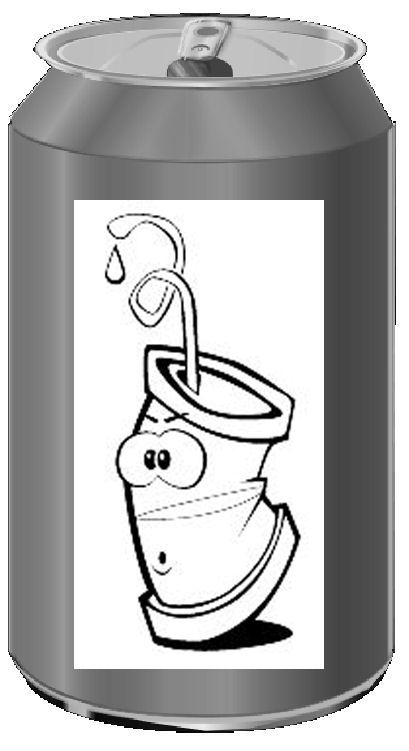

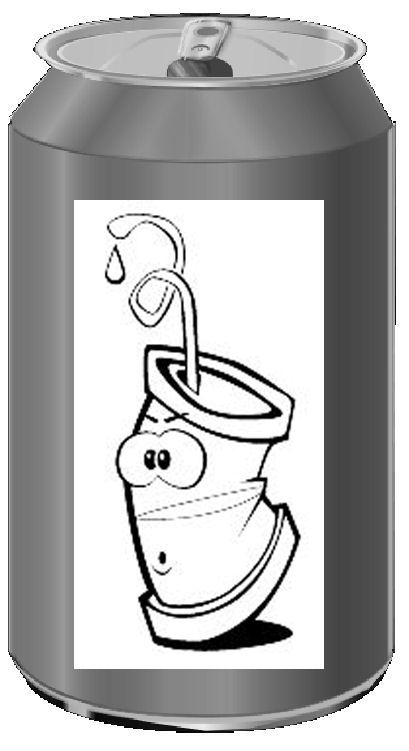

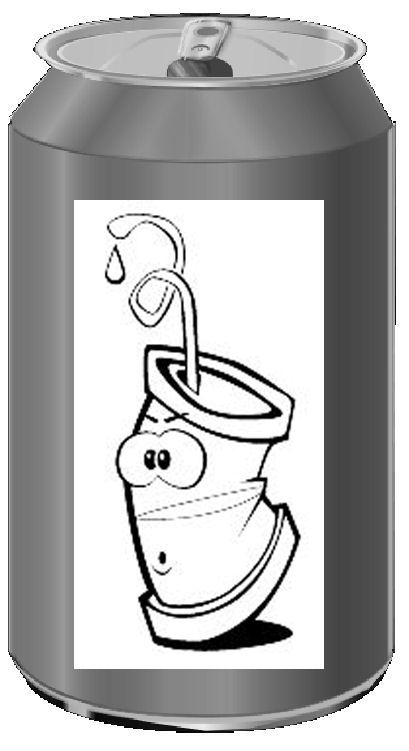

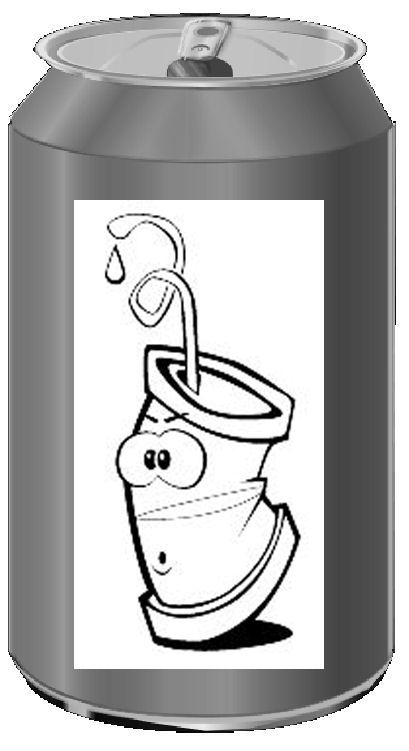
**Hoe vol zit je nu, VLAK VOORDAT je gaat eten en drinken?** *(How full do you feel, right before eating and drinking?)*

Ik heb nu **HEEL VEEL ZIN** in Blikkie *(I* ***really want*** *to drink Blikkie)*

Ik heb nu **BEST WEL ZIN** in Blikkie *(I* ***want*** *to drink Blikkie)*

Ik heb nu **EEN BEETJE ZIN** in Blikkie *(I* ***want*** *to drink Blikkie* ***a little bit****)*

Ik heb nu **GEEN ZIN** in Blikkie *(I* ***do not want*** *to drink Blikkie)*

Ik heb nu **HELEMAAL GEEN ZIN** in Blikkie *(I* ***really do not want*** *to drink Blikkie)*

BlikkieBlikkie.*would like to know if you want to drink Blikkie before you taste Blikkie. You tick the box that matches what you think)*

Wij willen weten hoe vol jij je voelt. Hieronder zie je 5 plaatjes van kinderen. Alle kinderen hebben een beetje eten in hun buik. Achter elk plaatje staat hoe het kind zich voelt. Zet een kruisje achter het kind dat zich net zo voelt als jij je **NU** voelt

*(We would like to know how full you feel. You see five pictures with children. All the children have some food in their stomach. Next to the picture we wrote the feeling of that child. You tick the box that matches what you feel)*

Ik voel me **HELEMAAL NIET VOL.** Ik zou nog heel veel kunnen eten *(I am* ***not full at all****. I could eat a lot)*

Ik voel me **EEN KLEIN BEETJE VOL**. Ik zou nog wel wat kunnen eten *(I am* ***a little bit full****. I could eat)*

Ik voel me **EEN BEETJE VOL**. Ik zou nog een beetje kunnen eten *(I am* ***a bit full****. I could eat a little bit)*

Ik voel me **BEST WEL VOL.** Ik zou nog een klein beetje kunnen eten *(I am* ***quite full****. I could eat only a very little bit)*

Ik voel me **HEEL VOL.** Ik zou niets meer kunnen eten *(I am* ***completely full.*** *I could not eat anymore)*

**EET & DRINKPAUZE***(Time to eat & drink)*

Nu mag je Blikkie opdrinken en gaan eten. We willen graag een paar dingen weten over het eten en drinken. De eerste vraag mag je met ja of nee beantwoorden

*(Now its time to drink Blikkie and eat your snack. We would like to know a few things about what you eat and drink. You can answer the first question with yes or no).*

**Eet je bij je BLIKKIE?** *(Are you eating while drinking your Blikkie?)*

Ja *(yes)*

Nee *(no)*

**Als je antwoord Nee was, ga je nu naar de volgende bladzijde. Als je antwoord Ja was, willen we nu precies weten wat je bij Blikkie eet. Je mag je antwoord schrijven op de stippellijn** *(If you answered with ‘no’, please go to the next page. If you answered with ‘yes’, we would like to know exactly what you are eating. Your can write your answer on the dotted line).*

**Bijvoorbeeld** (For example):

Wat eet je nu bij het drankje? Koekje

*(What are you eating with the beverage? Cookie)*Hoeveel eet je ervan? 2 stuks

*(How much are you eating? 2 pieces)*Welke naam staat er op de verpakking? Sultana naturel

*(Which name is printed on the packaging? Sultana natural)*

**Wat eet je nu bij het drankje?...............................................**

*(What are you eating with the beverage?)***Hoeveel eet je ervan?............................................................**

*(How much are you eating?)***Welke naam staat er op de verpakking?................................**

*(Which name is printed on the packaging?)*

**Hoe lekker vind je Blikkie?**

*(How much do you like Blikkie?)*

Nu je Blikkie opgedronken hebt, willen wij graag weten hoe lekker je Blikkie vindt. Hieronder zie je 5 gezichtjes. Zet een kruisje achter het gezichtje dat bij jou past

*(Now that you finished Blikkie, we would like to know how much you like Blikkie. You see five pictures with faces. You tick the box that matches what you feel)*

Ik vind Blikkie **heel erg lekker *(****I find Blikkie* ***delicious***

Ik vind Blikkie l**ekker**

*(I* ***like*** *Blikkie)*

Ik vind Blikkie **niet vies maar ook niet lekker** *(I* ***neither like nor dislike*** *Blikkie)*

Ik vind Blikkie **een beetje vies** *(I* ***do not like*** *Blikkie)*

Ik vind Blikkie **heel erg vies**

*(I find Blikkie* ***disgusting****)*

**Beantwoord nu de volgende vraag met ja of nee.**

*(Answer the following question with ‘yes’ or ‘no’)*

**Heb je BLIKKIE helemaal leeg gedronken?**

*(Did you finish the whole can?)*

**Ja** *(yes)*

**Nee** *(no)*

**Hoe vol zit je nu, DIRECT NADAT je gegeten en gedronken hebt?** *(How full do you feel, directly after eating and drinking?)*

**NU JE BLIKKIE OPGEDRONKEN HEBT**, willen wij weten hoe vol jij je voelt. Zet een kruisje achter het kind dat zich net zo voelt als jij je **NU** voelt.

*(Now that you finished Blikkie, we would like to know how full you feel. You tick the box that matches what you feel)*

Ik voel me **HELEMAAL NIET VOL**. Ik zou nog heel veel kunnen eten. *(I am* ***not full at all****. I could eat a lot)*

Ik voel me **EEN KLEIN BEETJE VOL**. Ik zou nog wel wat kunnen eten. *(I am* ***a little bit full****. I could eat)*

Ik voel me **EEN BEETJE VOL.** Ik zou nog een beetje kunnen eten. *(I am* ***a bit full****. I could eat a little bit)*

Ik voel me **BEST WEL VOL**. Ik zou nog een klein beetje kunnen eten. *(I am* ***quite full.*** *I could eat only a very little bit)*

Ik voel me **HEEL VOL**. Ik zou niets meer kunnen eten. *(I am* ***completely full.*** *I could not eat)*

**NU HEB JE PAUZE** *(Break time)*

**
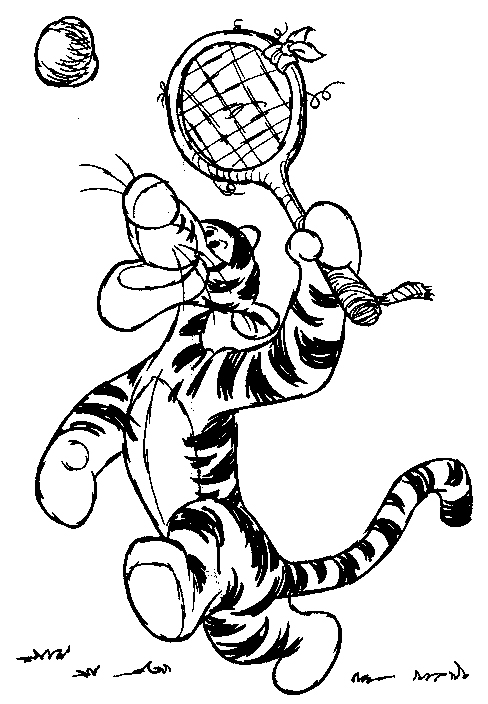
DIRECT NA DE PAUZE***(Right after the break)*

Nu je pauze hebt gehad, willen we graag weten hoe vol jij je **NU** voelt. Zet een kruisje achter het kind dat zich net zo voelt als jij je **NU** voelt.

*(Now that the break is over, we would like to know how full you feel. You tick the box that matches what you feel)*

Ik voel me **HELEMAAL NIET VOL**. Ik zou nog heel veel kunnen eten. *(I am* ***not full at all****. I could eat a lot)*

Ik voel me **EEN KLEIN BEETJE VOL** Ik zou nog wel wat kunnen eten. *(I am* ***a little bit full****. I could eat)*

Ik voel me **EEN BEETJE VOL.** Ik zou nog een beetje kunnen eten. *(I am* ***a bit full****. I could eat a little bit)*

Ik voel me **BEST WEL VOL** Ik zou nog een klein beetje kunnen eten. *(I am* ***quite full****. I could eat only a very ittle bit)*

Ik voel me **HEEL VOL.** Ik zou niets meer kunnen eten. *(I am* ***completely full****. I could not eat anymore)*

**Dank je wel voor het invullen!
Je kunt deze vragenlijst nu inleveren bij je juf of meester**

*(Thank you for filling in the questionnaire. Please give the questionnaire to the teacher)*
